# Multistable switches and their role in cellular differentiation networks

**DOI:** 10.1186/1471-2105-15-S7-S7

**Published:** 2014-05-28

**Authors:** Ahmadreza Ghaffarizadeh, Nicholas S Flann, Gregory J Podgorski

**Affiliations:** 1 Computer Science Department, Utah State University, Logan, UT 84322, USA; 2 Biology Department, Utah State University, Logan, UT 84322, USA; 3 Institute for Systems Biology, Seattle, WA 98109, USA; 4 Synthetic Biomanufacturing Institute, Logan, UT 84322, USA; 5 Center for Integrated BioSystems, Utah State University, Logan, UT 84322, USA

## Abstract

**Background:**

Cellular differentiation during development is controlled by gene regulatory networks (GRNs). This complex process is always subject to gene expression noise. There is evidence suggesting that commonly seen patterns in GRNs, referred to as biological multistable switches, play an important role in creating the structure of lineage trees by providing stability to cell types.

**Results:**

To explore this question a new methodology is developed and applied to study (a) the multistable switch-containing GRN for hematopoiesis and (b) a large set of random boolean networks (RBNs) in which multistable switches were embedded systematically. In this work, each network attractor is taken to represent a distinct cell type. The GRNs were seeded with one or two identical copies of each multistable switch and the effect of these additions on two key aspects of network dynamics was assessed. These properties are the barrier to movement between pairs of attractors (separation) and the degree to which one direction of movement between attractor pairs is favored over another (directionality). Both of these properties are instrumental in shaping the structure of lineage trees. We found that adding one multistable switch of any type had a modest effect on increasing the proportion of well-separated attractor pairs. Adding two identical switches of any type had a much stronger effect in increasing the proportion of well-separated attractors. Similarly, there was an increase in the frequency of directional transitions between attractor pairs when two identical multistable switches were added to GRNs. This effect on directionality was not observed when only one multistable switch was added.

**Conclusions:**

This work provides evidence that the occurrence of multistable switches in networks that control cellular differentiation contributes to the structure of lineage trees and to the stabilization of cell types.

## Introduction

Understanding differentiation is critical to knowing how normal development unfolds and for taming diseases, such as cancer, that are associated with defects or reversals in differentiation. In animals, the process of differentiation typically results in cells reaching a terminally differentiated state. However, recent discoveries have shown that "terminal differentiation" may be a misnomer as fully differentiated cells can be reprogrammed to revert back to a pluripotent state, with these pluripotent cells having the potential to differentiate into other cell types.

Transitions between cell types can be mapped as a directed tree of cell types, known as a lineage tree, with embryonic stem cells at the root, various classes of precursor cells as internal nodes, and terminally differentiated cells as branch tips. Gene regulatory networks (GRNs) that respond to both external stimuli and to gene expression noise control transitions between cell types and determine the structure of lineage trees [1]. Given that differentiation is driven by the output of dynamic gene regulatory networks, a useful, network-based perspective for envisioning different stable cell types is as basins in an attractor landscape [2,3]. In this dynamical systems view, differentiation is the process of moving between the different attractor basins that are generated by the dynamics of the gene regulatory network.

The GRNs that control differentiation are complex, but these larger networks can be decomposed into smaller modules of simpler, frequently appearing regulatory motifs that consist of only a few genes that interact in characteristic patterns[1]. For example, a common feature of many regulatory motifs is a pair of genes coupled by either positive or negative feedback loops [4]. These couplings result in different network outputs, with positive feedback loops often producing two or more stable attractor states, and negative feedback loops often enhancing attractor stability [4]. The generation of two or more attractors is referred to as multistability, with the special case of generating only two attractors termed bistability.

In this work, we investigated four regulatory motifs, termed multistable switches, that operate in differentiating cells[4,1]. Each of these motifs results in multistability when the motif operates in isolation[1]. These multistable switches were added singly or in identical pairs to larger GRNs to understand how they affect the structure of lineage trees and the stability of different cell types. These studies were done by generating random Boolean GRNs that produce five or more attractors.

These networks were then seeded with the multistable switches. We found that the addition of identical pairs multistable switches of any of the four different types increased the stability of attractors produced by the GRNs. Adding a single multistable switch of any type had little effect on attractor stability. The addition of two multistable switches to a randomly generated GRN also increased the proportion of directional transitions between attractors. In terms of differentiation, this contributes to the structure of a lineage tree by favoring particular pathways that lead between different cell types.

## Approach and results

This work studied three key properties of cellular differentiation[5]: (a) differentiation of multipotent cells can be driven by gene expression noise; (b) there is a strong directionality to differentiation, with transitions between cell types occurring from less to more differentiated cells; and (c) terminally differentiated cells are stable.

**Figure 1:**
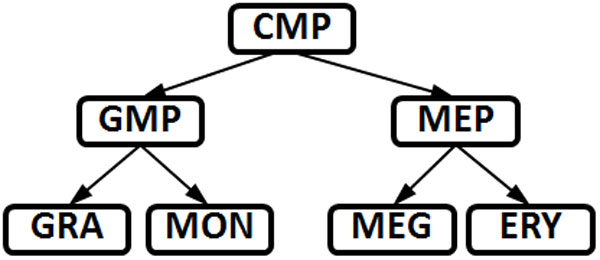
**A simplified myeloid lineage tree**. A simplified myeloid lineage tree (from [11]) where the terminal nodes are the mature cell types of erythrocytes (ERY), megakaryocytes (MEG), monocytes (MON), and granulocytes (GRA). Multipotent cells are the common myeloid progenitor (CMP), megakaryocyte-erythrocyte progenitor (MEP), and granulocyte-monocyte progenitor (GMP).

The simplified myeloid linage tree illustrated in Figure 1 provides an example of these key properties. This lineage tree includes only favored transitions between cell types that involve progenitor cells giving rise to two different, more differentiated cell types, and the establishment of barriers between cell types that prevent transdifferentiation and dedifferentiation.

### Cellular differentiation and attractor dynamics

**Figure 2:**
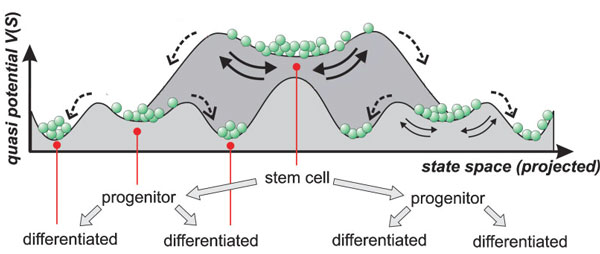
**A hypothetical two dimensional epigenetic landscape of differentiation (modified from **[1]**)**. The horizontal axis shows the state space of different cell types and the vertical axis approximates potential energy differences between cell types. The basins are attractors that represent different cell types and the magnitude of potential energy differences between states provides a measure of the probability of transitions between states under gene expression noise.

In this work, differentiation is viewed as a set of transitions between attractor basins produced by a dynamical genetic regulatory network. This model of differentiation was pioneered by Kaufman and extended by many others [6,3,7,5]. Borrowing from early work by Waddington[8], the landscape created by these attractor basins has been termed an epigenetic landscape [1]. A conceptual model of such an epigenetic landscape is shown in Figure 2. In this view, each cell type occupies an attractor basin at a particular level of a potential energy landscape. A cell can be moved out its attractor basin in response to an external signal or to gene expression noise. Once it crosses the barrier that delimits the basin, it moves down to another attractor basin lower in the epigenetic landscape. There are at least two possible paths leaving each attractor basin, with each downhill path leading to a different basin that represents a distinct, more specialized cell type. Once a cell descends into a new basin, the large potential energy barrier between the new lower basin and upper starting basin makes it unlikely for a more specialized cell to make the transition back to a progenitor cell. This process of cells moving out of an attractor basin in response to external signals or to gene expression noise and descending into attractor basins of lower potential energy that correspond to more differentiated cells is repeated at each level of the lineage tree.

This potential energy barrier that must be crossed to move between attractor basins is called the epigenetic barrier. Schmulevich et al. [9] proposed a method of quantifying this barrier termed the mean first passage time (MFPT), defined as the average number of state transitions needed to move from one attractor basin to another during the noisy operation of a Boolean regulatory network. The MFPT provides a measure of the probability of a particular transition between two attractor basins, with low MFPTs indicating a high likelihood of the transition, and high MFPTs indicating a low likelihood for this transition. Details on the calculation of MFPT values and all other aspects of the procedures are given in Methods; this section will only provide an overview.

The forward and reverse MFPT values between two attractor basins (simply called attractors from this point forward), *att*_1 _and *att*_2_, provide information on the directionality of the transition. Directionality is a key element of differentiation, as under normal circumstances, cells transition from less to more mature states, but not in the reserve direction. For the pair of attractors *att*_1 _and *att*_2_, we define a **directional transition **to occur if *att*_1 _*→ att*_2 _(reaching *att*_2 _from *att*_1_) has a significantly larger MFPT than the MFPT of *att*_2 _*→ att*_1_.

Another important aspect of cellular differentiation captured by MFPT is the probability of making a transition between any pair of different cell types. This is important in shaping the structure of a lineage tree and in stabilizing cell types. For example, progenitor cell types should not differentiate into cell types off the normal lineage path, and terminally differentiated cells must be prevented from dedifferentiation or transdifferentiating into other cell types. Therefore, the MFPT should be high in both directions for unfavored transitions between attractors. We term this **separation**, with high separation occurring when the MFPTs of *att*_1 _*→ att*_2 _and *att*_2 _*→ att*_1 _are both large.

**Figure 3:**
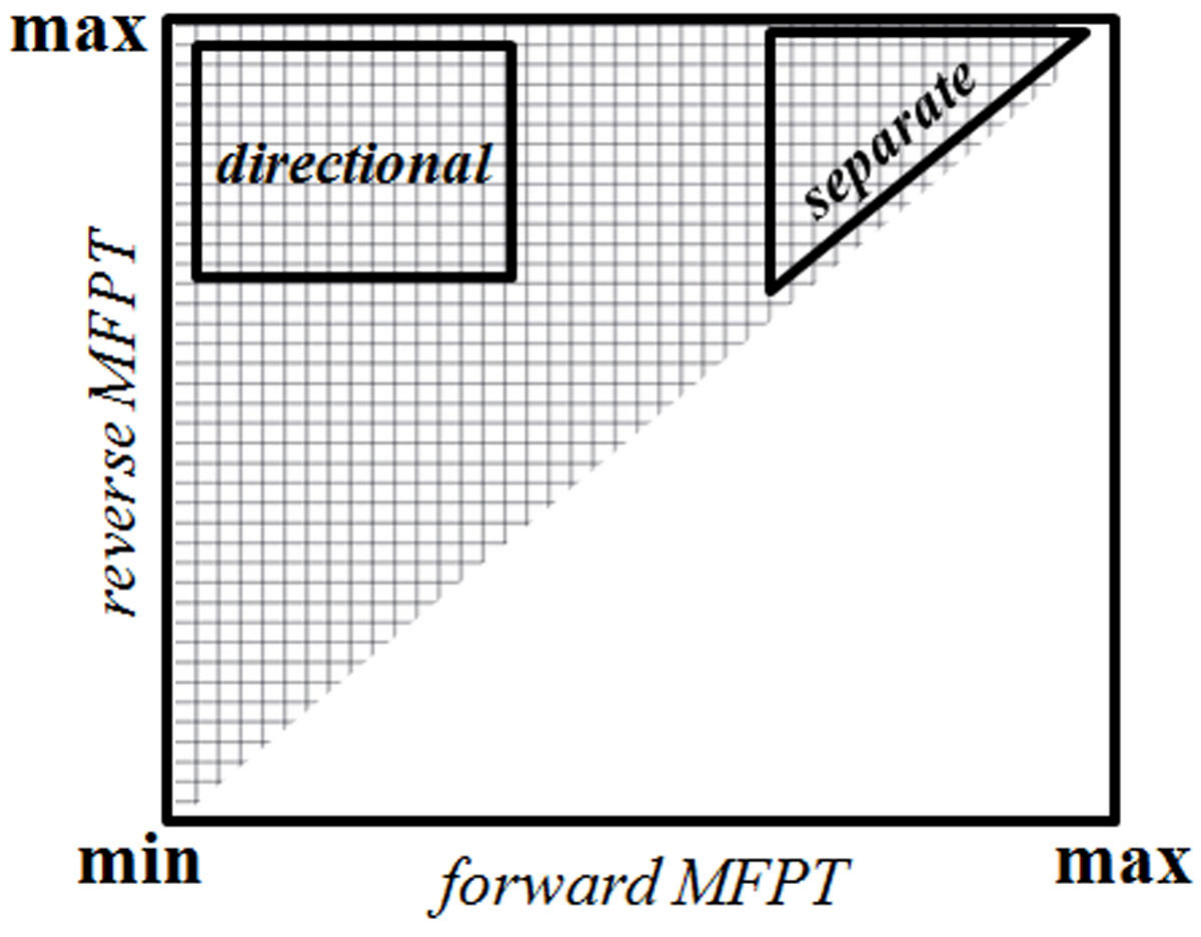
**Regions representing desired properties of a differentiation tree**. Forward and reverse MFPT plot showing *directional *and *separate *regions.

Given the directionality of differentiation and the large separation of the majority of cell types within a linage tree, a plot of the distribution of MFTPs of the forward (for example, *att*_1 _*→ att*_2_) and reverse (*att*_2 _*→ att*_1_) transitions between all possible pairs of cell types within a lineage tree is expected to show clustering in the regions of directionality and separation. This is shown in Figure 3. In this plot of forward and reverse MFPTs between all possible attractor pairs produced by a set of gene regulatory networks, the quadrant with a low forward MFPT and high reverse MFPT represents attractor pairs (cell types) that are linked with a strong directional transition. In contrast, the quadrant with high MFTPs in both the forward and reverse directions represents well separated attractor pairs. This region of high separation represents low probability transitions between cells types, such as transdifferentiation or differentiation off the normal lineage pathway. Using this reasoning, if adding a small multistable switch to a larger GRN enhances the directionality of transitions between attractors, then in a plot like the one shown in Figure 3, there should be an increase in frequency of attractor pairs in the regions labeled *directional*. Similarly, if a multistable switch added to a gene regulatory network increases the separation between pairs of attractors, then there should be an increase in the region of Figure 3 labeled *separate*. This is the basis of the approach followed in this work.

An important point to note is that in a MFPT representation of biologically realistic lineage trees, the proportion of attractor pair transitions in the *separate *region will far exceed the proportion in the directional quadrant. This is because the topology of actual linage trees leads to there being significantly fewer directional transitions than well separated transitions. Intuitively, this stems from the ideas that the number of favored transitions between different cell types is much smaller than the number of theoretically possible transitions, and that most of the theoretically possible transitions are unfavored events such as dedifferentiation and transdifferentiation. Mathematically, the possible number of well separated transitions is on the order *O*(*b*^2*h*^) while the number of directional transitions is of the order *O*(*b^h^*), where *b *is the branching factor of differentiation tree (number of children for each node) and *h *is the height of the tree measured as the number of cell type transitions between a stem cell and a terminally differentiated cell. This expected difference in the proportions of *separate *and *directional *attractor pair transitions is important when interpreting the effects of adding multistable switches to random Boolean genetic regulatory networks (see below).

**Figure 4:**
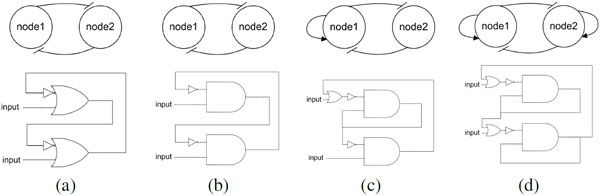
**Multistable switches used in this work**. The diagrams in the left show the node and edge representation and the diagrams at the right show the logic gate representation of each switch. The truth table of the functions are [1,1,0,1] for *a *and [0,1,0,0] for *b, c*, and *d *for binary numbers [00,01,10,11], respectively. In this work, the multistable switches are referred to as: (a) bistable switch (*BS *), (b) mutual inhibition with zero positive feedback loops (*MI00*), (c) mutual inhibition with one positive feedback loop (*MI0+*), and (d) mutual inhibition with two positive feedback loops (*MI++*).

We investigated how the addition of the four multistable switches shown in Figure 4 influenced the attractor landscape produced by randomly generated Boolean regulatory networks. A conventional node-and-edge diagram of each multistable switch used in biological literature is depicted in the figure, followed by a more informative logic circuit representation. The first logic circuit (Figure 4.a) is usually referred to as a bistable switch (BS) or toggle switch[10]. We call the second logic switch (4.b) a mutual inhibition switch (MI00). Note how the less informative node-and-edge diagrams for these two distinct logic circuits are identical. The next two multistable switches extend mutual inhibition with the addition of one (*MI+0*) or two (*MI++*) positive feedback loops. *MI++ *is sometimes referred to as tristable switch.

### Multistable switches in myeloid differentiation

An important example of cellular diversification is the well studied system of hematopoiesis. During hematopoiesis, multipotent stem cells (hemocytoblasts) differentiate into either myeloid or lymphoid progenitors [11]. A sub-tree of the myeloid lineage tree is illustrated in Figure 1. This figure shows that common myeloid progenitor (CMP) cells produce two pluripotent cell types (megakaryocyte-erythrocyte progenitor (MEP) cells and granulocyte-monocyte progenitor (GMP) cells) that in turn produce terminally differentiated erythrocyte, megakaryocyte, monocyte and granulocyte cells.

**Figure 5:**
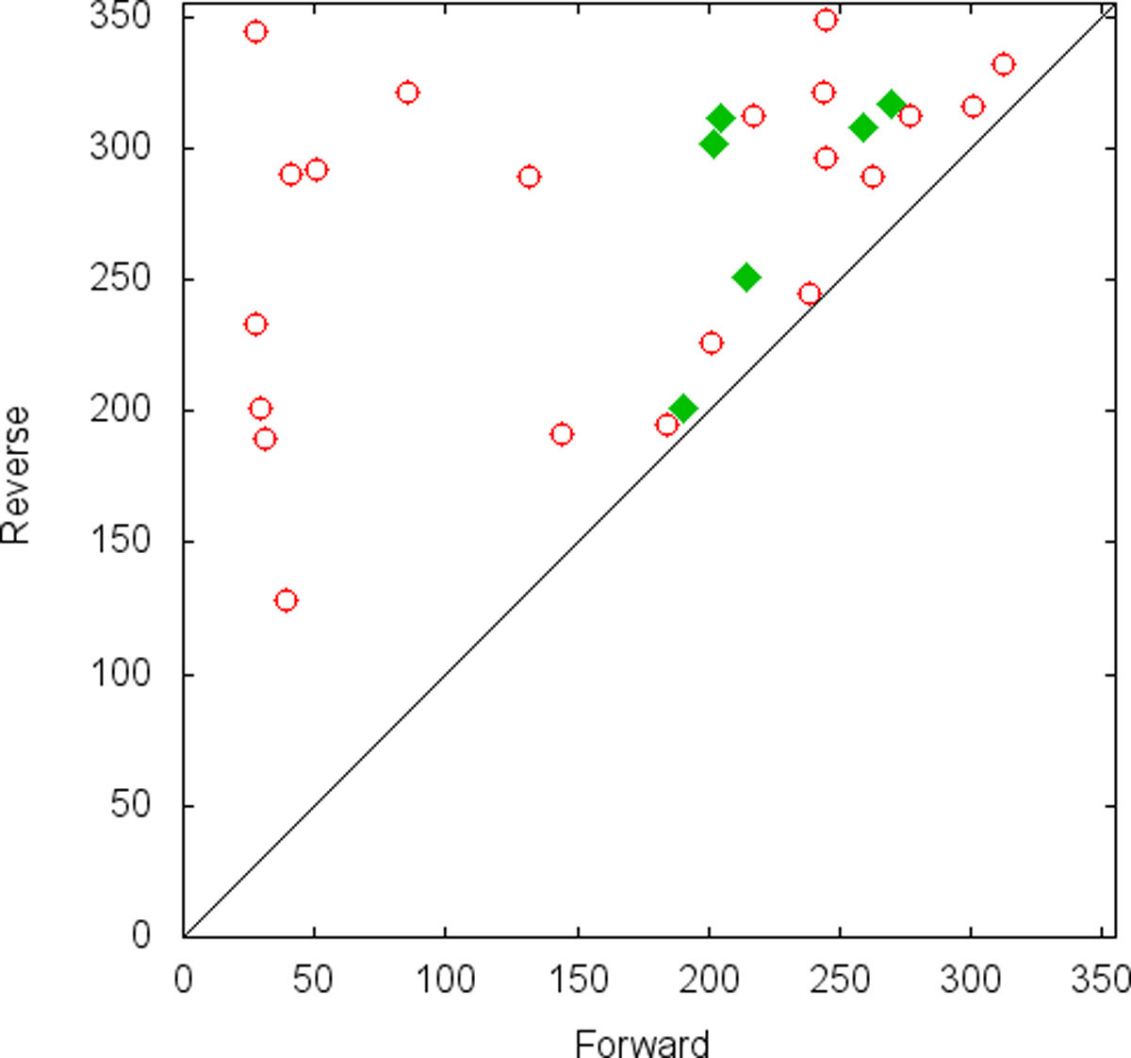
**Forward and reverse MFPT plot for the myeloid differentiation network**. Red circles are the MFPT values of our inferred network. This network has all 7 attractors of the myeloid lineage tree shown in Figure 1, including multipotent cells. Green diamonds show the MFPT values for the network proposed by Krumsiek et al. which only has the 4 terminally differentiated cell types[11]. Including multipotent cells illustrates additional attractor relations, including directionality.

To construct a GRN that simulates the dynamics of the myeloid differentiation, we extracted a set of regulatory gene expression levels of all cell types in Figure 1 from three datasets of distinct experiments available at ArrayExpress database (http://www.ebi.ac.uk/microarray-as/ae/): E-GEOD-5606, E-GEOD-8407, and E-GEOD-18483. Motivated by Krumsiek et al. [11], we picked 11 transcription factors that play important roles in myeloid differentiation: GATA-1, GATA-2, FOG-1, EKLF, Fli-1, SCL, C/EBP*α*, PU.1, cJun, EgrNab, and Gfi-1; note that the EgrNab, represents an integration of Egr-1, Egr-2 and Nab-2. Using these genes and their expression profiles, we utilized a search tool to infer a GRN for myeloid differentiation as a Boolean network (manuscript in preparation). This network includes 4 well-known gene interactions that represent multistable switches [11], [1]: a) An MI++ switch between GATA-1 and PU.1; b) An MI++ switch between GATA-2 and PU.1; c) A bistable switch between Fli-1 and EKLF; and d) A bistable switch between Gfi-1 and EgrNab. We computed the MFPT between attractors of this network that represent the cell types of the myeloid lineage tree. The pairwise forward and reverse MFPT values between all pairs of attractors of this network are depicted in the Figure 5 (red circles); we also included the MFPT values for the attractors of the original network proposed by Krumsiek and colleagues (green diamonds) that contains only four attractors as the terminally differentiated cell types. This figure shows that the majority of transitions in myeloid differentiation fall in either the separation or directionality regions shown in Figure 3.

### Multistable switches in random networks

We showed that the myeloid differentiation network, with its multistable switches, generates directional transitions and well separated attractors. How general is this result? We extended our study to examine the role of these switches in a large space of cellular differentiation networks.

The outline of this approach was to:

1 Construct a random Boolean network (only networks that are expected to operate in the critical domain were generated (see Methods)).

2 Embed zero, one or two copies of a given multistable switch within the network.

3 Run the network and identify attractors; if the number of attractors is less than 5, go back to step 1.

4 Compute the forward and reverse MFPT between all pairs of attractors.

5 Map the forward and reverse MFPT of each pair of attractors to a point in a MFPT density plot like the one shown in Figure 3.

6 Repeat for 5000 random Boolean networks to create each MFPT density plot.

**Figure 6:**
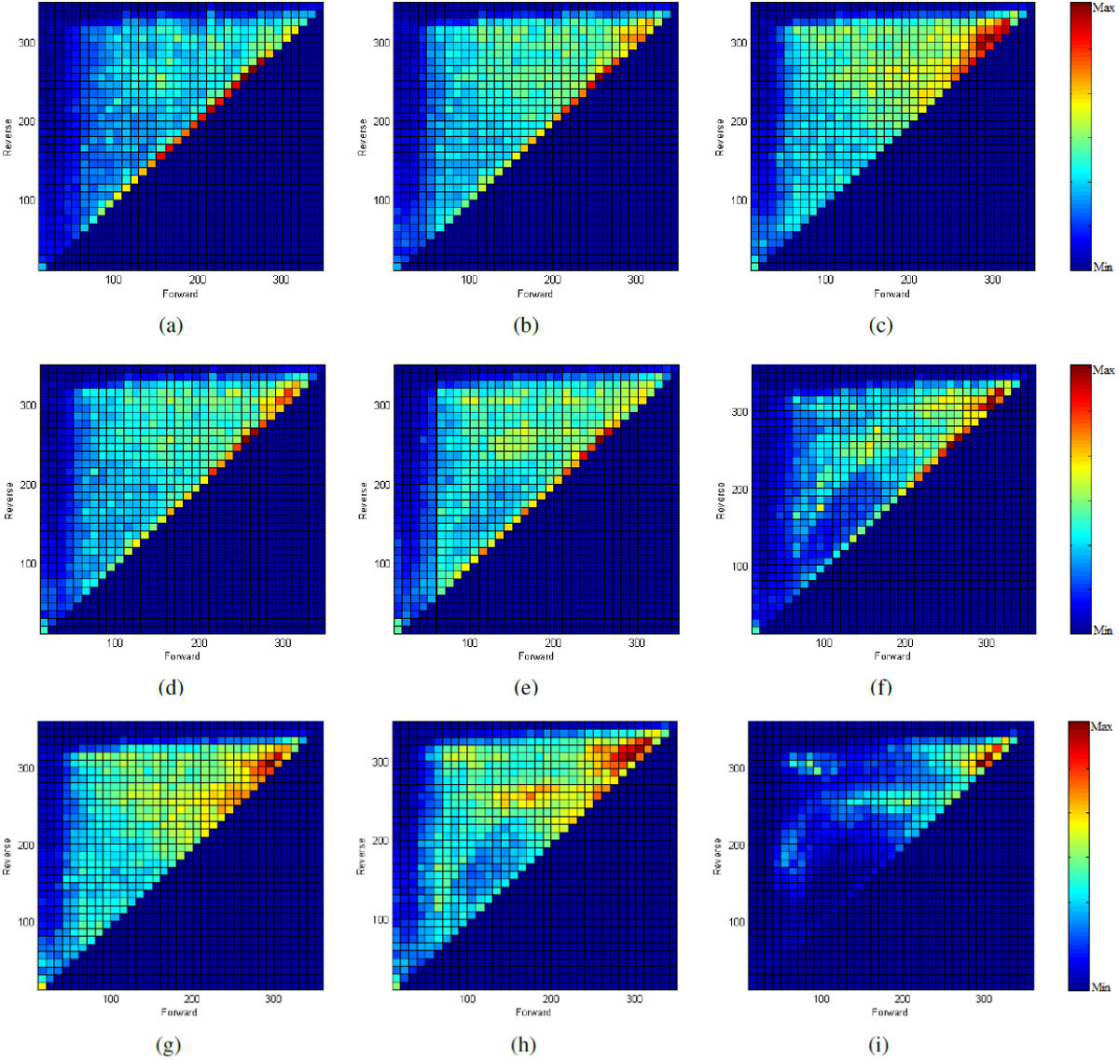
**Distributions of MFPT values**. The plots show the forward and reverse MFPTs for all transitions seen in 5000 critical networks of each type. (a) Networks with no added multistable motifs; (b) Networks with one embedded bistable switch; (c) Networks with two embedded bistable switches; (d) Networks with one embedded MI00 switch; (e) Networks with one embedded MI+0 switch; (f) Networks with one embedded MI++ switch; (g) Networks with two embedded MI00 switches; (h) Networks with two embedded MI+0 switches; (i) Networks with two embedded MI++ switches.

Density plots were generated for 9 different types of networks: RBN networks without any added multistable switch and RBNs with one or two identical copies of each of the four types of multistable switches. Figure 6 shows these density plots. Each plot shows the forward and reverse MFPT between all attractor pairs generated by 5000 networks of a single type.

The MFPT density distribution produced by RBNs without any added multistable switch (Figure 6a) shows no clustering in the *directional *or *separate *regions of the plot. Instead, the forward and reverse MFPTs of most of the transitions are equal and of intermediate values and therefore fall in the mid-range of the diagonal. Adding a single multistable switch of any type to the RBN had a modest effect of increasing the density of attractor pairs in the *separate *region. Adding two multistable switches of the same type to the RBN had a much stronger effect on increasing the frequency of well separated attractor pairs. This is reflected in an increased density in the *separate *region of the MFPT plots. The particular kind of multistable switch had little impact on this effect; instead, the critical element was adding two rather than one multistable switch to the RBN.

**Figure 7:**
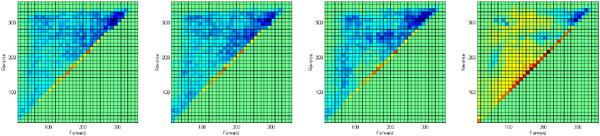
**Difference of distributions of MFPT values**. Difference of distributions of MFPT values for networks embedded with two identical motifs against the networks with no motifs. (a) Difference of network with no motifs and networks with two embedded bistable switches; (b) Difference of network with no motifs and networks with two embedded MI00 switches; (c) Difference of network with no motifs and networks with two embedded MI+0 switches; (c) Difference of network with no motifs and networks with two embedded MI++ switches.

There was a modest increase in the density of attractor pairs in the *directional *regions of the MFPT plot when two identical multistable switches were added. However, as discussed above, a major clustering of MFPT values in the *directional *region is not expected in networks that produce lineage trees. The modest increase in directionality gained by adding multistable switches is likely to be significant. In contrast to the effect on separation, there was a difference between the multistable switch types in increasing directionality: The MI++ switch type did not increase directionality, but all three of the other types did. To better illustrate these enrichments in *directional *and *separate *regions, Figure 7 shows the difference between the MFPT distribution of networks with two embedded multistable switches and the base-line random network distribution.

## Conclusion

This work examined how the attractor structure generated from random Boolean regulatory network dynamics was influenced by the addition of multistable switches that are commonly found in biological networks that control differentiation. The results show that the addition of multistable switches increases the resilience of genetic regulatory networks to gene expression noise. This is seen by the increase in the proportion of well separated attractors. In a biological context, this separation of attractors has the effect of stabilizing determined cells and of helping to establish well defined pathways between differentiating cells. Adding a single multistable switch to a random network had a relatively modest stabilizing effect, but adding two identical switches of any of the four types tested here produced much stronger barriers between different cell types. In parallel, there was also evidence that adding two multistable switches to a genetic regulatory network increased the frequency of directional transitions between attractors. From a biological perspective, this structures a linage tree by favoring one-way transitions between particular cell types. Therefore, the pervasive occurrence of multistable switches in networks that control cellular differentiation is likely to contribute to the structure of lineage trees and to the stabilization of cell types.

## Detailed methods

### Cell differentiation and attractor dynamics

Boolean networks [6] have proved effective in representing GRN structure and dynamics in many systems, including *Drosophila *development [12,13], angiogenesis [14], eukaryotic cell dynamics [15], and yeast transcription networks [16]. Each gene in a network is represented as a node whose regulation by other genes is modeled using updating rules as logic functions. An expressed gene is assigned the value true and a non-expressed gene the value false.

A Boolean network with *n *genes has 2*^n ^*possible states, denoted as *Ŝ*. At each step in the simulation, the next state *ŝ*_*t*+1 _∈ *Ŝ *is determined by applying each gene's logic function (representing the regulatory interactions) to the current value of the genes in *ŝ_t_*. Let this computation be defined as *ŝ*_*t*+1 _*← D*(*ŝ_t_*) where *D*(*ŝ_t_*) is the deterministic mapping function that finds the next state of the network given the current state. As the network is executed by repeated applications of *D*(*ŝ_t_*), the state will reach a previously visited state, and thus, since the dynamics are deterministic, enter into an attractor which represents a fixed point of the system. Attractors can be single states, called point attractors, or consist of more than one state that the network continuously transitions between, called cyclic attractors. Let *â *= *D*^∗^(*ŝ*) be the resulting network attractor state reached when starting at *ŝ *and applying the logic functions until the attractor state *â *is reached.

In this work, cell types are considered attractors in the state space of possible gene expression profiles [17] and cell differentiation is modeled as the process of transitioning from one attractor to another [18].

### Network construction

A random Boolean regulatory network is generated by randomly connecting a varying number of nodes, then instantiating each node with a randomly generated logic function. To replicate networks found in natural systems, we created only networks that operate in the critical domain, rather than ordered or chaotic. Critical networks implement maximal information flow [3] and have the lowest attractor basin entropy [19]. Evidence that GRN's tend to be critical is given in [20]. To generate critical networks, the parameters are set according to *s *= 2*qp_N_*(1 − *p_N_*) where *s *is the sensitivity of the network to perturbations in gene values, *p_N _*is the probability of the output of each Boolean function being 1, and *q *is the count of inputs to each Boolean function [21]. When *s *= 1 a single bit change is on average propagated to one other node and the network is in the critical domain. In an ordered network, *s *< 1 and perturbations tend to die out, while in a chaotic network, *s *> 1 and perturbations tend to grow. In this work *s *was fixed at 1 and *p_N _*was adjusted depending upon the value of *q*.

**Figure 8:**
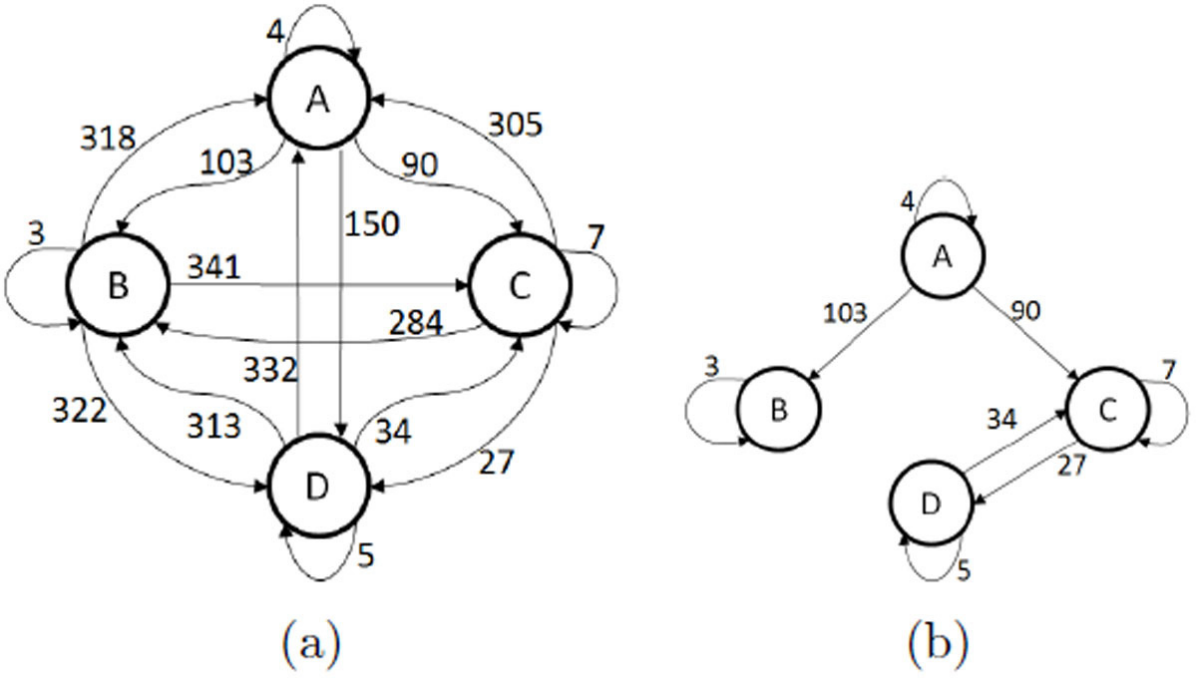
**Mean first passage time graphs**. (a) A sample MFPT graph. Nodes are attractors and the weights of edges are proportional to MFPT values between attractors. (b) Same graph as in (a) with high (> 103) MFPT edges eliminated.

The attractors of each random Boolean regulatory network are determined, then Markov chain analysis is performed to determine the transition probabilities between all possible states. This allows determination of the MFPTs between each pair of attractors [9]. The MFPTs allow the construction of a graph whose nodes are attractors and weighted edges are the MFPT value between different nodes. Figure 8.a shows a sample graph. MFPT graphs for cellular differentiation are expected to have a small MFPT value for forward edges (moving from less to more specialized cell types), large values for reverse edges, and large values in both directions for transitions between attractors at the same level of tree (level is the number of transitions from the root). In [5] a method was introduced that applied successively higher MFPT thresholds to prune edges from this complete MFPT tree as a means to identify separation among subsets of close attractor states as illustrated in Figure 8(b). The effects of changing the threshold from low to high was proposed as a possible mechanism for cellular differentiation with the low threshold representing pluripotency and the process of raising the threshold as type specialization as attractors become more and more isolated. This model proposes that cells differentiate by actively controlling their sensitivity of expression noise and can account for the observation that terminally differentiated cell states tend to be more stable than pluripotent states.

### Network search

We perform a uniform Monte Carlo search over the space of critical random Boolean networks. For each network we find the attractors and compute the MFPT between all possible attractor pairs (extended from code posted at http://code.google.com/p/pbn-matlab-toolbox[9]). Using the MFPT values, for each type of multistable switch added to the network, we draw a density plot where the x-axis is the forward MFPT and the y-axis is the reverse MFPT (we consider the edge with lower MFPT as forward). The acquired density plots are used to determine the distribution of directional, non-directional, separated and non-separated probability transitions between attractors in each network.

### Network types

We investigated nine types of networks. Approximately 5 ∗ 10^4 ^networks of each type were explored to find 5, 000 networks of each type with five or more attractors. The different network types come from the use of the 4 multistable switches that are shown in Figure 4. The first switch (Figure 4 a) is a bistable switch, a small local circuit with feedback loops. This is a common switch in biological networks and it controls binary branch points between two mutually exclusive cell lineages[1,10]. The truth table of the functions in this switch is [1,1,0,1] for binary numbers [00,01,10,11] respectively. The other three switches all encode mutual inhibition between two genes. The first is *MI00 *and is based on the network synthesized in [10]. *MI00 *includes two incoherent feedback loops. The final two switches extend mutual inhibition with the addition of one *MI+0 *or two *MI++ *positive (coherent) feedback loops. These two switches were explored in [22], where it was shown that the positive feedback loops can introduce additional shallow attractor basins in continuous ODE network models.

The four switches were used as described above to construct nine different types of networks: no motif, one BS, one MI00, one MI0+, one MI++ and then four more network classes each with two of the same switch. Note that when a motif defined in Figure 4 is embedded, two nodes of the original RBN are selected randomly, their logic functions replaced and inputs and outputs rewired. For illustration, consider how a MI0+ motif is embedded into a RBN. Starting with a RBN (see Figure 4 (a)), two nodes are selected randomly and their truth tables are changed to [0,1,0,0]. Then, the 2^0 ^input of the second node is wired to the output of first node and, conversely, the 2^0 ^input of first node is wired to the output of the second node. The small or-gate and not-gate are not considered in wiring, because they were previously considered in the truth tables of their respective nodes.

### Mean first passage time

The first-passage time (*FPT*), also called first hitting time, is the time taken by a stochastic system for the first visit of a specific state. Mathematically, *FPT *is defined as *F_k_*(*ŝ_x_, ŝ_y_*): the probability that starting in state $\hat{x}$, the first time the system visits a state *ŷ *will be at time *k*. In the case of Boolean networks, time is the path length of state transitions. Considering *p_xy _*as the probability of transition between states *x *and *y*, then *F*_1_(*ŝ_x_, ŝ_y_*) = *p_xy_*. As equation 1 shows, for *k *≥ 2, *F_k _*is calculated by a recursive iteration over all transitive relations: for all *z *states in the network dynamics, *F_k_*(*ŝ_x_, ŝ_y_*) is the probability of a one step transition from state *x *to *z *times the *FPT *from state *z *to *y *in *k − *1 steps.

(1) $$F_{k}\left({\hat{s}}_{x},{\hat{s}}_{y}\right)=\underset{{\hat{s}}_{z}\in {\left\{0,1\right\}}^{n},z\neq y}{\sum }p_{xz}F_{k-1}\left({\hat{s}}_{z},{\hat{s}}_{y}\right)$$

Probabilistically, there are two possibilities to reach state *y *from *x*; either *y *is a deterministic target for *x *and no bit flips occur due to the noise, or an aggregate of bit flips drive the transition from *x *to *y*. So the equation for *p_xy _*can be written as follows.

(2) $$pxy=\left\{\begin{array}{cc} {\left(1-p_{e}\right)}^{n} & y\leftarrow D\left({\hat{s}}_{x}\right)\\ p_{e}^{h_{xy}}{\left(1-p_{e}\right)}^{n-h_{xy}} & y\leftarrow \eta \left({\hat{s}}_{x},h_{xy}\right),{\hat{s}}_{x}\neq {\hat{s}}_{y} \end{array}\right)$$

where *d_ij _*is equal to 1 if there is a deterministic transition from *x *to *y *in the network dynamics, otherwise it is 0; *p_e _*is the probability of a single bit flip resulting from noise and *h_xy _*is the Hamming distance between two states; *n *is the total number of nodes in the network.

Although the *FPT *is a valuable measure, the average time it takes to reach state *y *from state *x*, termed Mean First Passage Time (*MFPT*), is of greater interest. MFPT in Boolean networks was introduced by Shmulevich et al [23] and is defined as:

(3) $$MFPT\left({\hat{s}}_{x},{\hat{s}}_{y}\right)=\underset{k}{\sum }kF_{k}\left({\hat{s}}_{x},{\hat{s}}_{y}\right)$$

**Figure 9:**
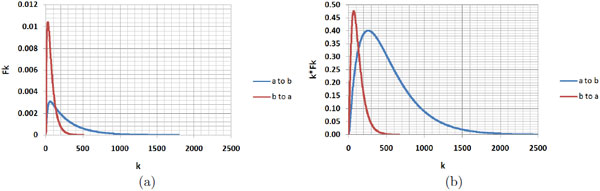
**Illustration of mean first passage time**. (a) *F_k _*(probability of first visit at time step *k*) plotted for two arbitrary attractors, called *a *and *b*, in a random Boolean network for 2500 steps (*k*). The red curve is for the transition from *a *to *b *that has a low MFPT compared to the reverse transition, *b *to *a *(shown with the blue curve); (b) *kF_k _*plotted for the *F_k _*curves in (a). Note that MFPT is the centroid of area under the *kF_k _*curve.

A low *MFPT *between two states indicates that starting from the first state, the second state is easily reached by gene expression noise. Figure 9 shows *F_k_, kF_k_*, and MFPT for the transition between two arbitrary attractors. As this figure shows, the *a *to *b *transition has a lower MFPT compared to the other.

At each network state update *D*(*ŝ*) there is a probability that the state will change as a function of the Hamming distance (*h*) between the current state and the subsequent state *ŝ*_*t*+1 _← *D*(*η*(*ŝ_t_, r*)). *MFPT *models uniform gene expression noise by considering probabilistic bit flips at every possible state of the network and deriving the distribution of passage times from analysis of the corresponding Markov process. Statistically, the probability distribution of bit flips can be seen as a binomial distribution, thus the probability of *r *bit flips, *η*(*ŝ_a_, r*) is $\left(\begin{array}{c} h\\ r \end{array}\right)p^{r}{\left(1-p\right)}^{h-r}$, where *p *is the probability of a single bit flip and *h *is the total number of bits.

## Comparisons of epigenetic barrier measures

There are a number of possible ways to measure epigenetic barriers that separate two attractor basins. In this part of the work, the utility of three of these measures, MFPT, transitory bit flips, and Hamming distance, were compared.

### Evaluating epigenetic barriers: MFPT vs. transitory bit flips

Villani et al. [5] studied noise-driven network transitions in RBNs. They introduced a measure of the probability of network transitions as the likelihood of attractor transition under expression noise. In this measure, for each pair of attractors {*a_i_, a_j_*}, *P*(*i, j*) is the portion of single one-step bit flips (transitory perturbations) in the nodes of all states of attractor *a_i _*which will result in a transition from *a_i _*to *a_j _*under noise-free dynamics. The measure of likelihood of network transition under noise is similar to MFPT, but it does not consider gene expression variability throughout the network. MFPT better models global expression noise by considering probabilistic bit flips at every possible state of the network and deriving the distribution of passage times from analysis of the corresponding Markov process.

**Figure 10:**
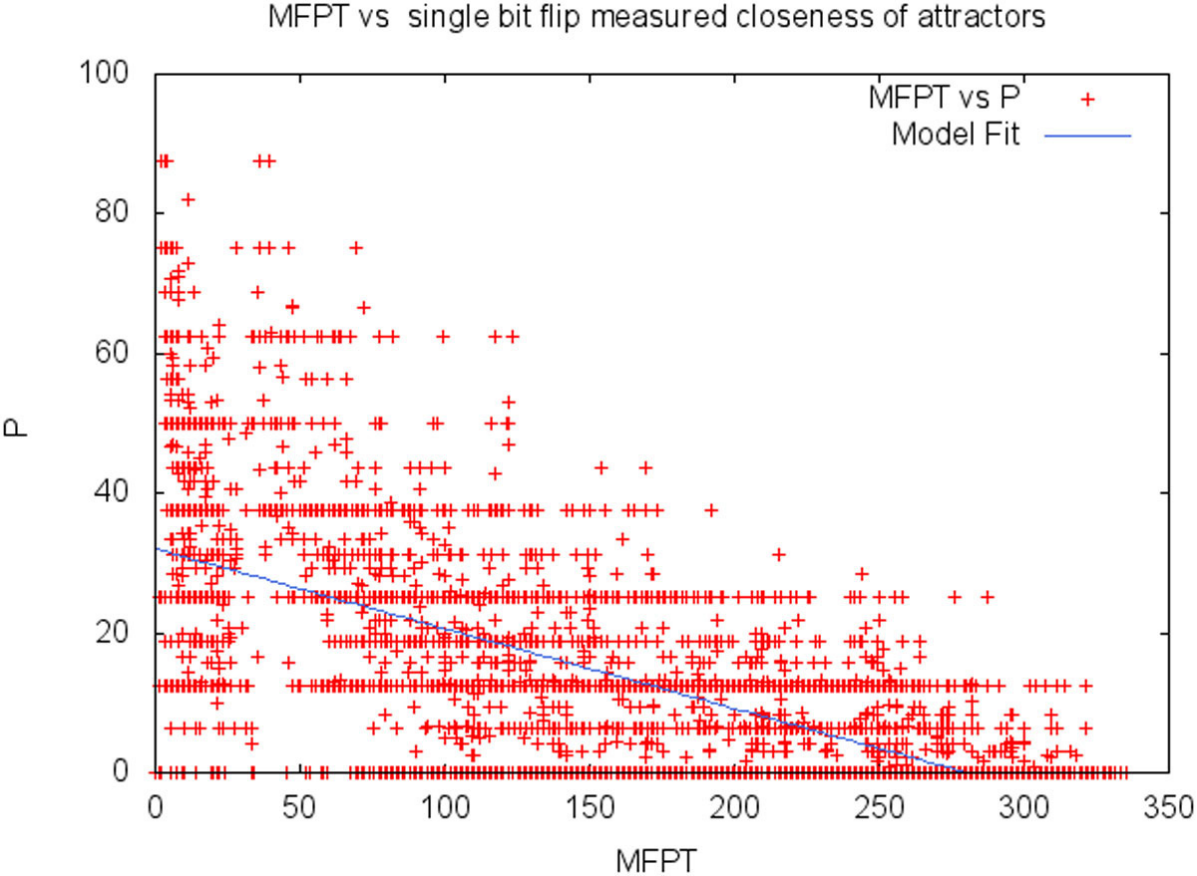
**MFPT vs. transitory bit flips**. Relationship between MFPT and *P *for 100 critical RBNs.

Since one-off bit flips consider noise only as a single bit changes and only when the network has reached its attractor states, it could serve as an efficient yet heuristic measure of the MFPT. To test this idea, a study was performed on a set of small critical networks where for each network and each pair of attractors, *P*(*i, j*) was compared with MFPT(*i, j*). Figure 10 depicts the relationship between MFPT and *P *for 100 arbitrary Boolean networks that have 5 or more attractors. Each point represents the epigenetic barrier between two attractors measured in MFPT and *P*. Since the networks studied in these experiments are small and do not have many attractors, many points are located in the line *P *= 0. The regression line in this figure shows that as MFPT increases *P *tends to decrease. *P *and MFPT are modestly correlated for these small networks and it is unclear how well one-off bit flips can accurately estimate MFPT when network size grows. Since the networks in our experiments are small, we only consider MFPT because of its realism in modeling expression noise.

### Evaluating epigenetic barriers: MFPT vs. Hamming distance

**Figure 11:**
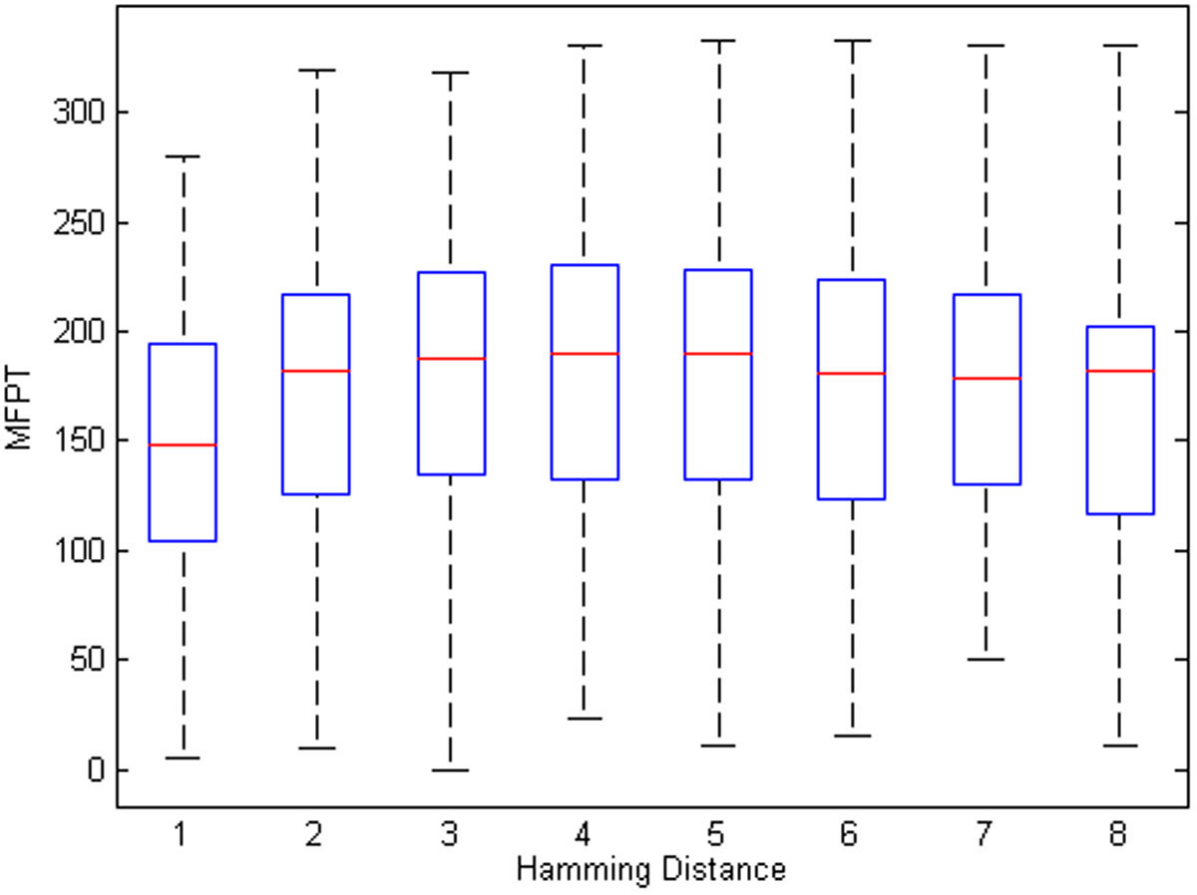
**MFPT vs. Hamming distance**. Relationship between average MFPT between attractor pairs and Hamming distance for 100 critical BNs.

An intuitive idea is that MFPT between attractors has a direct relationship to the Hamming distance that separates these attractors. However, we found that this is not the case. Instead, network dynamics, not the Hamming distance, is the main contributor to the MFPT between attractors. As an example of the limitations of Hamming distance, consider that the MFPT(*a_i_, a_j_*) and MFPT(*a_j_, a_i_*) can be different, but that the Hamming distance between these attractors is the same. However, even though there is not a strong relationship between MFPT and Hamming distance, a weak correlation between the average of the forward and reverse MFPT between attractors and their Hamming distance can be detected. This is depicted in Figure 11 which shows MFPT versus the Hamming distance obtained from 100 RBNs containing 8 nodes. As the Hamming distance increases, the upper-bound of MFPT values also increases (*r *= 0.1027 for Hamming distance and average MFPT). In Figure 11, the box represents the central 50% of the points and the red bar shows the median of data.

## Competing interests

The authors declare that they have no competing interests.

## Authors' contributions

AG, NF and GP conceived the system, designed experiments and wrote the text. AG performed all the experiments.

## Declarations

This work was supported by the Luxembourg Centre for Systems Biomedicine, the University of Luxembourg and the Institute for Systems Biology, Seattle, USA. Research reported in this publication was partially supported by the National Institute Of General Medical Sciences of the National Institutes of Health under Award Number P50GM076547. The content is solely the responsibility of the authors and does not necessarily represent the official views of the National Institutes of Health.

This article has been published as part of *BMC Bioinformatics *Volume 15 Supplement 7, 2014: Selected articles from the 10th Annual Biotechnology and Bioinformatics Symposium (BIOT 2013). The full contents of the supplement are available online at http://www.biomedcentral.com/bmcbioinformatics/supplements/15/S7
